# Histone deacetylase 7 activates 6-phosphogluconate dehydrogenase via an enzyme-independent mechanism that involves the N-terminal protein-protein interaction domain

**DOI:** 10.1042/BCJ20240380

**Published:** 2024-10-25

**Authors:** Yizhuo Wang, James E.B. Curson, Divya Ramnath, Kaustav Das Gupta, Robert C. Reid, Denuja Karunakaran, David P. Fairlie, Matthew J. Sweet

**Affiliations:** 1 Institute for Molecular Bioscience (IMB), The University of Queensland, Brisbane, Queensland 4072, Australia; 2 Australian Infectious Diseases Research Centre, The University of Queensland, Brisbane, Queensland 4072, Australia; 3 Victorian Heart Institute, Victorian Heart Hospital, Clayton, Victoria 3168, Australia; 4 Department of Physiology, Monash Biomedicine Discovery Institute, Monash University, Clayton, Victoria 3800, Australia; 5 ARC Centre of Excellence for Innovations in Peptide and Protein Science, Institute for Molecular Bioscience, The University of Queensland, Brisbane, Queensland 4072, Australia

**Keywords:** 6-phosphogluconate dehydrogenase, HDACs, histone deacetylase 7, inflammation, lysine deacetylase, scaffolding

## Abstract

Histone deacetylase 7 (HDAC7) is a member of the class IIa family of classical HDACs with important roles in cell development, differentiation, and activation, including in macrophages and other innate immune cells. HDAC7 and other class IIa HDACs act as transcriptional repressors in the nucleus but, in some cell types, they can also act in the cytoplasm to modify non-nuclear proteins and/or scaffold signalling complexes. In macrophages, HDAC7 is a cytoplasmic protein with both pro- and anti-inflammatory functions, with the latter activity involving activation of the pentose phosphate pathway (PPP) enzyme 6-phosphogluconate dehydrogenase (6PGD) and the generation of anti-inflammatory metabolite ribulose-5-phosphate. Here, we used ectopic expression systems and biochemical approaches to investigate the mechanism by which HDAC7 promotes 6PGD enzyme activity. We reveal that HDAC7 enzyme activity is not required for its activation of 6PGD and that the N-terminal protein-protein interaction domain of HDAC7 is sufficient to initiate this response. Mechanistically, the N-terminus of HDAC7 increases the affinity of 6PGD for NADP^+^, promotes the generation of a shorter form of 6PGD, and enhances the formation of higher order protein complexes, implicating its scaffolding function in engagement of the PPP. This contrasts with the pro-inflammatory function of HDAC7 in macrophages, in which it promotes deacetylation of the glycolytic enzyme pyruvate kinase M2 for inflammatory cytokine production.

## Introduction

As sentinel cells of the innate immune system, macrophages sense danger via cell surface and intracellular receptors to respond to different stimuli. Recognition of lipopolysaccharide (LPS) from Gram-negative bacterial cell walls by toll-like receptor 4 (TLR4), for example, initiates multiple signalling pathways to alter cell metabolism, inflammatory pathways, and host defence responses. These complex signalling cascades are initiated and tuned by multiple enzymes that initiate post-translational modifications [1], including regulated lysine acetylation [2]. Histone deacetylases (HDACs) are lysine deacetylases that control epigenetics and gene expression in the nucleus but also have important roles in signal transduction in the cytoplasm [2]. HDAC7, a member of the class IIa subfamily of classical HDACs, carries out both these functions in different cell types, with wide-ranging roles in physiological and pathological processes [3].

The class IIa HDACs (including HDAC4, 5, 7 and 9) share sequence similarity, containing a highly conserved C-terminal zinc-binding deacetylase domain and a less conserved N-terminal domain that can interact with various non-histone proteins [4,5]. This subfamily is generally considered enzymatically inactive due to the lack of a key active site tyrosine residue that is present in other classical HDACs. In class IIa HDACs, this residue is instead a histidine, with this key difference generally thought to be responsible for greatly reduced enzymatic activity [6]. However, some evidence suggests that class IIa HDACs may function as active enzymes, rather than exclusively as acetyl-lysine docking proteins. For example, HDAC7 controls macrophage metabolism and inflammatory responses [7,8], promoting LPS-inducible inflammatory responses via deacetylation of the glycolytic enzyme pyruvate kinase isoform M2 (PKM2) at K433 to enable HIF-1α dependent transcriptional responses [8]. Notably, HDAC7 also binds to HDAC3, with this interaction contributing to HDAC7's enzyme activity in non-macrophages [9]. HDAC3 also regulates inflammatory responses [10], so it is possible that this class I HDAC may also be involved in the HDAC7-PKM2-HIF-1α axis in macrophages, although this has not been investigated. Apart from its involvement in this inflammatory pathway, the proteomic screen that revealed an association between HDAC7 and PKM2 also identified the pentose phosphate pathway (PPP) enzyme 6-phosphogluconate dehydrogenase (6PGD) as a candidate HDAC7-interacting partner [8]. Subsequent investigations revealed that HDAC7 has distinct functions in macrophages depending on the nature of the activating stimulus, with particulate matter such as whole bacteria triggering HDAC7-dependent activation of 6PGD for suppression of specific inflammatory responses in macrophages [11].

6PGD is the third enzyme in the PPP. Branching off glycolysis, the PPP regulates redox homeostasis and supports fatty acid synthesis via an oxidative branch and maintains cell growth and proliferation via a non-oxidative branch [12,13]. By consuming the glycolytic product, glucose 6-phosphate, the oxidative branch of the PPP generates ribulose-5-phosphate (RL5P) and two molecules of nicotinamide adenine dinucleotide phosphate (NADPH). RL5P enters the non-oxidative branch, being converting to ribose-5-phosphate that is required for nucleotide synthesis [12]. NADPH is required to generate reactive oxygen species (ROS) and glutathione [14,15], thus playing an import role in controlling cellular redox status. This process is mediated by two NADPH-producing enzymes, glucose-6-phosphate dehydrogenase (G6PD) and 6PGD [16,17], with 6PGD being the second NADPH-producing enzyme in the PPP. In phagocytic cells such as neutrophils and macrophages, the PPP fuels production of ROS that is used to directly target engulfed bacteria, thus contributing to antimicrobial defence [18]. The oxidative PPP and 6PGD activity are also implicated in cancer initiation, tumour growth and metastasis [19]. For example, genetic and pharmacological targeting of 6PGD suppressed the proliferation of various cancer cells and decreased tumour size *in vivo* [20,21]. The active form of 6PGD functions as a homodimer [22] and post-translational modifications that increase its activity, for example lysine acetylation [23] and tyrosine phosphorylation [24], increase cancer cell proliferation and tumour growth in mouse models.

We previously reported that in murine macrophages responding to bacteria, HDAC7 engages the PPP to generate NADPH and cellular ROS for bacterial killing [11]. The engagement of this pathway by HDAC7 also suppressed inflammatory responses, likely to enable macrophages to focus resources on antimicrobial defence. That study also showed that HDAC7 interacted with and activated 6PGD, with its enzymatic product, RL5P, exerting both anti-inflammatory and antimicrobial effects. By contrast, acetylation of 6PGD at K76 and K294 enhanced its enzyme activity in human H1299 cancer cells, promoting cell proliferation [23]. The study also identified both HDAC4 and HDAC7 as deacetylases of 6PGD; however, only HDAC4 immunoprecipitated from H1299 cell lysates decreased acetylation levels and enzymatic activity of recombinant 6PGD, with immunoprecipitated HDAC7 having no effect on these read-outs. Collectively, these findings suggest that there may be context-dependent effects of HDAC7 in controlling 6PGD activation. In this study, we used ectopic expression in HEK293 cells and *in vitro* assays to investigate mechanisms by which murine HDAC7 activates 6PGD.

## Results

### HDAC7 interacts with and activates 6PGD independently of its deacetylase domain

**Figure 1.**
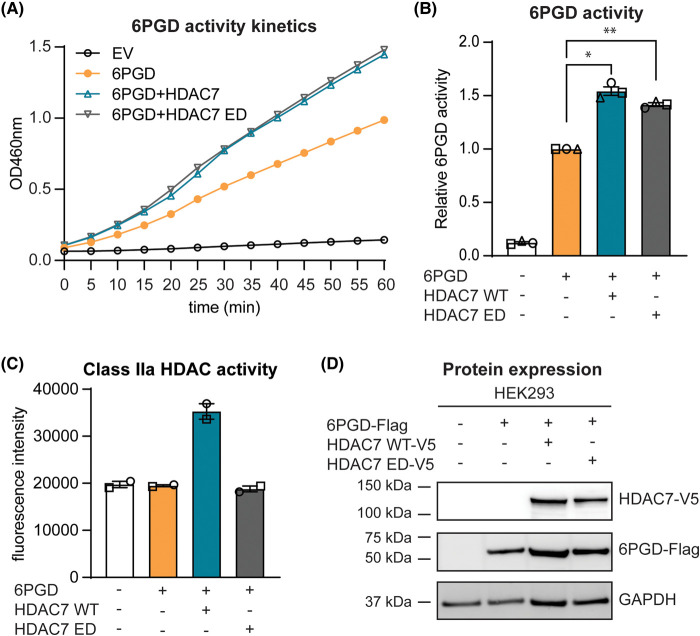
HDAC7 enzyme activity is not required to increase 6PGD enzyme activity. HEK293 cells were transfected with an empty vector (EV) control or expression constructs encoding 6PGD (Flag-tagged) and HDAC7 wild type (WT) or HDAC7 enzyme-dead (ED) (V5-tagged) at a molar ratio of 1:1 (6PGD:HDAC), after which the enzymatic activity of 6PGD, class IIa HDAC activity and protein expression in cell lysates was measured. (**A**) Kinetics of 6PGD enzymatic activity. Cell lysates were incubated with 6PGD substrate at 37°C for 60 min, with 6PGD enzyme activity being measured every 5 min. Results shown are from one representative experiment of three independent biological repeats. (**B**) 6PGD enzymatic activity in HEK293 cells. Absorbance at two timepoints from the linear phase of the kinetic curves (**A**) was used to calculate ΔOD, which was then applied to the standard curve generated using NADPH to determine 6PGD activity (nmol/min/ml). Data (mean ± SEM) are combined from three independent experiments and are displayed as relative activity (normalised to levels in cells transfected with 6PGD alone). Statistical analysis was performed using repeated measure one-way ANOVA followed by Dunnett's multiple comparison (*P < 0.05; **P < 0.01). (**C**) Class IIa HDAC enzymatic activity in transfected cells. Cell lysates were incubated with the class IIa HDAC fluorophore-conjugated substrate BOC-Lys(trifluoroacetyl)-AMC for 30–60 min at 37°C, after which stop reagent was added and fluorescence measured. Data (mean ± range) are combined from two independent experiments. (**D**) Protein expression of 6PGD (anti-Flag), HDAC7 WT and HDAC7 ED (anti-V5) in transfected HEK293 cells was assessed by immunoblotting, with GAPDH used as a loading control. Data shown are representative blots, with similar results apparent in three independent experiments.

Considering that HDAC7 can deacetylate non-histone protein substrates [3] and that its enzymatic activity mediates pro-inflammatory responses in macrophages [8,25], we wondered whether its enzyme activity is also required for activation of 6PGD. To examine this, we overexpressed a Flag-tagged 6PGD expression construct in HEK293 cells along with constructs encoding either V5-tagged wild type HDAC7 (WT) or an enzyme dead (ED) HDAC7 mutant, which has an inactivating mutation in the enzymatic domain (H649A) [8], and then measured 6PGD activity in the transfected cells. In comparison to cells transfected with 6PGD alone, both WT and ED HDAC7 increased the enzymatic activity of 6PGD to a similar level (Figure 1A,B). An *in vitro* class IIa HDAC activity assay was performed in parallel to confirm that the ED HDAC7 mutant was inactive in these assays (Figure 1C), with expression of all constructs being confirmed by immunoblotting for the Flag and V5 tags (Figure 1D). These data thus indicate that HDAC7 enzyme activity is not required for it to increase 6PGD enzyme activity in transfected HEK293 cells.

**Figure 2.**
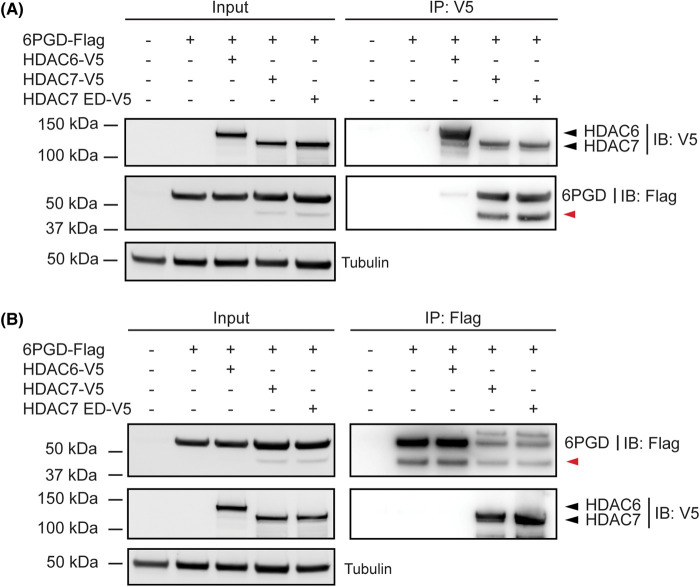
An enzyme-dead mutant of HDAC7 also interacts with 6PGD. HEK293 cells were transfected with 6PGD-Flag and HDAC7-V5, HDAC7 ED-V5 or HDAC6-V5 expression constructs at a molar ratio of 1:1 (6PGD:HDAC) for 24 h. Cells were then lysed and cell lysates were immunoprecipitated using an anti-V5 antibody (**A**) or an anti-Flag antibody (**B**), after which the interaction between 6PGD and HDAC7/HDAC6 was assessed by immunoblotting using antibodies against Flag and V5. Tubulin was also probed as a loading control. The displayed immunoblots are representative of two experiments (**A**) or are from a single experiment (**B**). The red arrow indicates a shorter form of 6PGD (6PGD-s) that was detected by anti-Flag immunoblotting.

Although 6PGD can be deacetylated at several lysine residues [23], the above data suggest that HDAC7 activates 6PGD through an enzyme-independent mechanism. Since the ED HDAC7 still activated 6PGD, we predicted that it would also associate with this PPP enzyme in cells. To test this, we assessed the ability of the ED HDAC7 mutant to immunoprecipitate 6PGD in transfected HEK293 cells (Figure 2). In contrast to an HDAC6-V5 negative control, both WT HDAC7 and the ED HDAC7 mutant strongly immunoprecipitated 6PGD in transfected HEK293 cells. These associations were apparent either when HDAC constructs were immunoprecipitated (anti-V5, Figure 2A) or when 6PGD was immunoprecipitated (anti-Flag, Figure 2B). Interestingly, a shorter ∼40 kDa form of 6PGD was observed in lysates and immunoprecipitates from cells co-transfected with either WT HDAC7 or ED HDAC7 (Figure 2, red arrows), which suggests that the ED HDAC7 may have the same effect as the WT HDAC7 in regulating 6PGD. The shorter form of 6PGD (hereafter 6PGD-s) was also more prominent in cells overexpressing HDAC7 than HDAC6 (Figure 2A,B, input). These data suggest a possible correlation between the presence of 6PGD-s and increased 6PGD enzyme activity.

### The N-terminal domain of HDAC7 is sufficient for 6PGD activation

**Figure 3.**
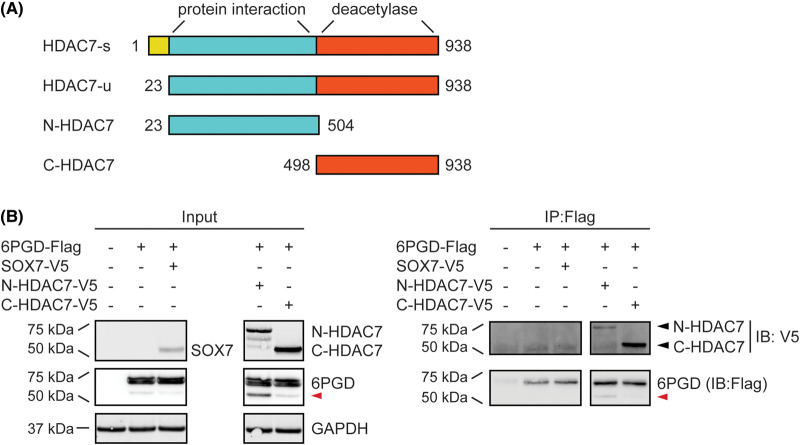
Both the N-terminal and C-terminal domains of HDAC7 interact with 6PGD in HEK293 cells. (**A**) Schematic diagram of HDAC7 constructs used for studying the interaction between HDAC7 and 6PGD. HDAC7-s, spliced, full-length HDAC7; HDAC7-u, unspliced isoform lacking the first 22 amino acids (NM_001204278.1); N-HDAC7, N-terminal domain of HDAC7; C-HDAC7, C-terminal domain of HDAC7. (**B**) Immunoprecipitation of 6PGD (anti-Flag). HEK293 cells were transfected with 6PGD-Flag and N-HDAC7-V5, C-HDAC7-V5 or SOX7-V5 (unrelated control) expression constructs at a molar ratio of 1:1 (6PGD:HDAC or SOX7) for 24 h. Cells were then lysed and cell lysates were immunoprecipitated using an anti-Flag antibody, after which the interaction between 6PGD and HDAC7 proteins was assessed by immunoblotting using antibodies against V5. GAPDH levels were assessed as a loading control. The displayed immunoblots are from one experiment that is representative of two independent experiments. The red arrow indicates a shorter form of 6PGD (6PGD-s) that was detected by anti-Flag immunoblotting.

While the ED HDAC7 mutant is catalytically inactive, the catalytic domain of HDAC7 could still retain the capacity to interact with acetyl-lysine-containing proteins, and play a non-enzymatic role in influencing 6PGD activation. We therefore next assessed whether the deacetylase-containing C-terminus (amino acid residue 498–938) of HDAC7 has the capacity to enhance 6PGD enzymatic activity. Two HDAC7 truncation mutants were used for this purpose. The mouse HDAC7 protein has two characterised isoforms, with the shorter unspliced isoform (HDAC7-u) lacking the first 22 amino acids [26] and selectively promoting inflammatory phenotypes by comparison to the longer spliced isoform (HDAC7-s) [7]. The WT HDAC7 construct used in the above experiments was the short HDAC7-u isoform that has a total of 916 amino acids. The N-terminal HDAC7 construct (amino acids 23–504, N-HDAC7) lacks the C-terminal catalytic domain but contains motifs that bind MEF2 and other interacting proteins [27,28], whereas the C-terminal construct (amino acid residue 498–938, C-HDAC7) contains only the catalytic domain (Figure 3A). Interestingly, 6PGD co-immunoprecipitated both the N-terminal and C-terminal HDAC7 truncation mutants but not an unrelated control protein (SOX7) in HEK293 cells; the interaction with the C-terminal HDAC7 domain being more pronounced (Figure 3B). This indicates that 6PGD can directly or indirectly associate with two distinct regions of HDAC7. Interestingly, 6PGD-s was enriched in immunoprecipitates from cells expressing N-HDAC7, but not C-HDAC7 (Figure 3B, red arrow).

**Figure 4.**
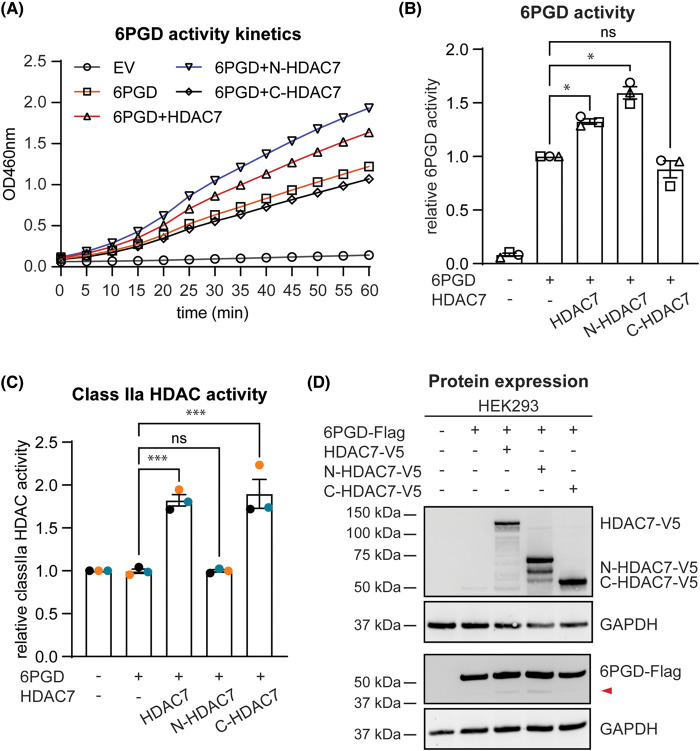
The N-terminal domain but not the deacetylase-containing C-terminal domain of HDAC7 enhances 6PGD enzyme activity. HEK293 cells were transfected with expression constructs encoding 6PGD (Flag-tagged) and full-length, N-HDAC7 or C-HDAC7 (V5-tagged) at a molar ratio of 1:1 (6PGD:HDAC) for 24 h, after which the enzymatic activity of 6PGD, class IIa HDAC activity and protein expression in cell lysates was measured. (**A**) Kinetics of 6PGD enzymatic activity. Cell lysates were incubated with the 6PGD substrate 6-phosphogluconate at 37°C for 60 min, with 6PGD enzyme activity being measured every 5 min. Results shown are from one representative experiment of three independent biological repeats. (**B**) 6PGD enzymatic activity in HEK293 cells. Absorbance at two timepoints from the linear phase of the kinetic curves (**A**) was used to calculate ΔOD, which was then applied to the standard curve generated using NADPH to determine 6PGD activity (nmol/min/ml). 6PGD activity was normalised to total protein concentration in cell lysates and data (mean ± SEM) are combined from three independent experiments, displayed as relative activity (normalised to levels in cells transfected with 6PGD alone). (**C**) Class IIa HDAC activity in cells transfected with the indicated constructs. Cell lysates were incubated with the class IIa HDAC-specific fluorophore-conjugated substrate BOC-Lys(trifluoroacetyl)-AMC for 30–60 min at 37°C, after which stop reagent was added and fluorescence measured. Data (mean ± SEM) are combined from three independent experiments (normalised to levels in cells transfected with EV). (**D**) Protein expression of 6PGD (anti-Flag) and HDAC7 (anti-V5) in transfected cells was assessed by immunoblotting, with GAPDH used as a loading control. Samples were analysed on two different SDS-PAGE gels to distinguish 6PGD and the truncated forms of HDAC7. The displayed immunoblots are representative of three independent experiments. The red arrow indicates a shorter form of 6PGD (6PGD-s) that was detected by anti-Flag immunoblotting. Statistical analyses (**B**,**C**) were performed using repeated measure one-way ANOVA followed by Dunnett's multiple comparison (ns, not significant; *P < 0.05; ***P < 0.001).

Since both domains of HDAC7 interacted with 6PGD (Figure 3B), we next investigated whether they could both increase 6PGD enzymatic activity. Here we observed that only the N-terminus of HDAC7 increased the enzymatic activity of overexpressed 6PGD, similar to the effect observed for the WT HDAC7 (Figure 4A,B). Lysates from cells transfected with WT HDAC7 or the C-HDAC7 truncation mutant displayed the expected class IIa HDAC enzymatic activity, whereas lysates from cells transfected with the N-HDAC7 construct did not (Figure 4C). Ectopic protein expression for all constructs was also confirmed by immunoblotting (Figure 4D). These data further confirm that HDAC7-mediated 6PGD activation does not require HDAC7 enzymatic function and revealed that the N-terminus of HDAC7 is sufficient for 6PGD activation in this cellular system.

### HDAC7 does not facilitate 6PGD dimerization

**Figure 5.**
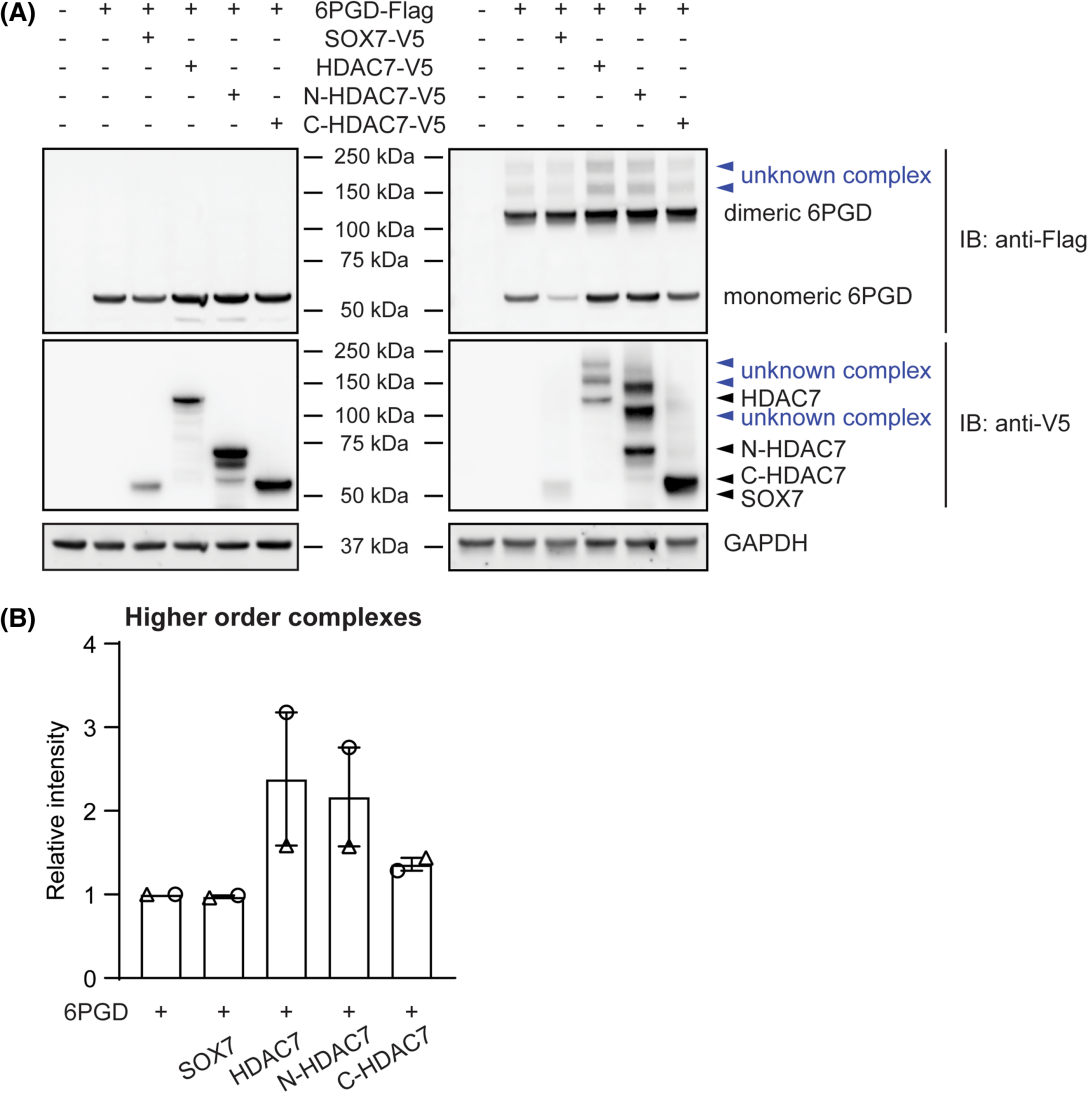
HDAC7 does not affect 6PGD dimerization in HEK293 cells but enhances the formation of 6PGD-containing higher order complexes. HEK293 cells were transfected with expression constructs encoding 6PGD (Flag-tagged) and full-length HDAC7, N-HDAC7, C-HDAC7 or SOX7 as an unrelated control protein (all V5-tagged) at a molar ratio of 1:1 (6PGD:HDAC7 or SOX7) for 24 h. (**A**) Cell lysates were immunoblotted (left) or were incubated with disuccinimidyl suberate to enable crosslinking of proteins, then immunoblotted (right). After the reaction was quenched by the addition of Tris-HCl, the same amounts of cell lysates from each transfection were used for immunoblotting to examine protein expression (left), as well as 6PGD dimerization and/or higher order complex formation (right). The levels of transfected proteins, including 6PGD monomers and dimers, were assessed by immunoblotting with antibodies against V5 and Flag, with GAPDH used as a loading control. Samples were analysed on the same SDS-PAGE gel, and fluorescent secondary antibodies were used to distinguish monomeric 6PGD and truncated HDAC7. The displayed immunoblots are from one experiment, representative of two independent experiments using different crosslinkers (similar results were obtained using glutaraldehyde as the crosslinker). (**B**) Quantification of the two higher order 6PGD-containing complexes across different transfection conditions. Data (displayed relative to the 6PGD alone control) are combined from two independent experiments and show the combined levels of the 150 and 200 kDa complexes.

Since the enzymatically active form of 6PGD is a homodimer [22], we next examined whether HDAC7 facilitates the dimerization of 6PGD to enhance its enzyme activity. To test this, we performed crosslinking assays using either disuccinimidyl suberate (DSS) or glutaraldehyde with lysates from cells transfected with expression constructs for Flag-tagged 6PGD and V5-tagged full-length HDAC7 or truncation mutants. The expression of transfected proteins was confirmed by immunoblotting against Flag or V5 tags using the non-crosslinked cell lysates (Figure 5A, left). Overexpression of WT HDAC7 or HDAC7 truncation mutants did not noticeably increase levels of dimeric 6PGD over monomeric 6PGD, as compared with the cells transfected with 6PGD alone (Figure 5A, right). This suggests that WT HDAC7 and N-HDAC7 do not strongly promote formation of dimeric 6PGD. However, overexpression of WT HDAC7 and N-HDAC7 did promote the formation of two higher order protein complexes of ∼150 and 200 kDa, respectively (Figure 5A,B). It is thus likely that both WT HDAC7 and N-HDAC7 facilitate formation of complexes that contain 6PGD and unknown interaction partners. Interestingly, higher order complexes were also apparent for WT HDAC7 and N-HDAC7 (Figure 5A, right), as assessed by probing the same cross-linked lysates with anti-V5 antibody. Overall, these results strongly implicate HDAC7, through its N-terminal domain, in promoting the formation of 6PGD-containing protein complexes rather than in enhancing the dimerization of 6PGD under the conditions employed here.

### HDAC7 enhances NADP^+^ affinity of 6PGD

**Figure 6.**
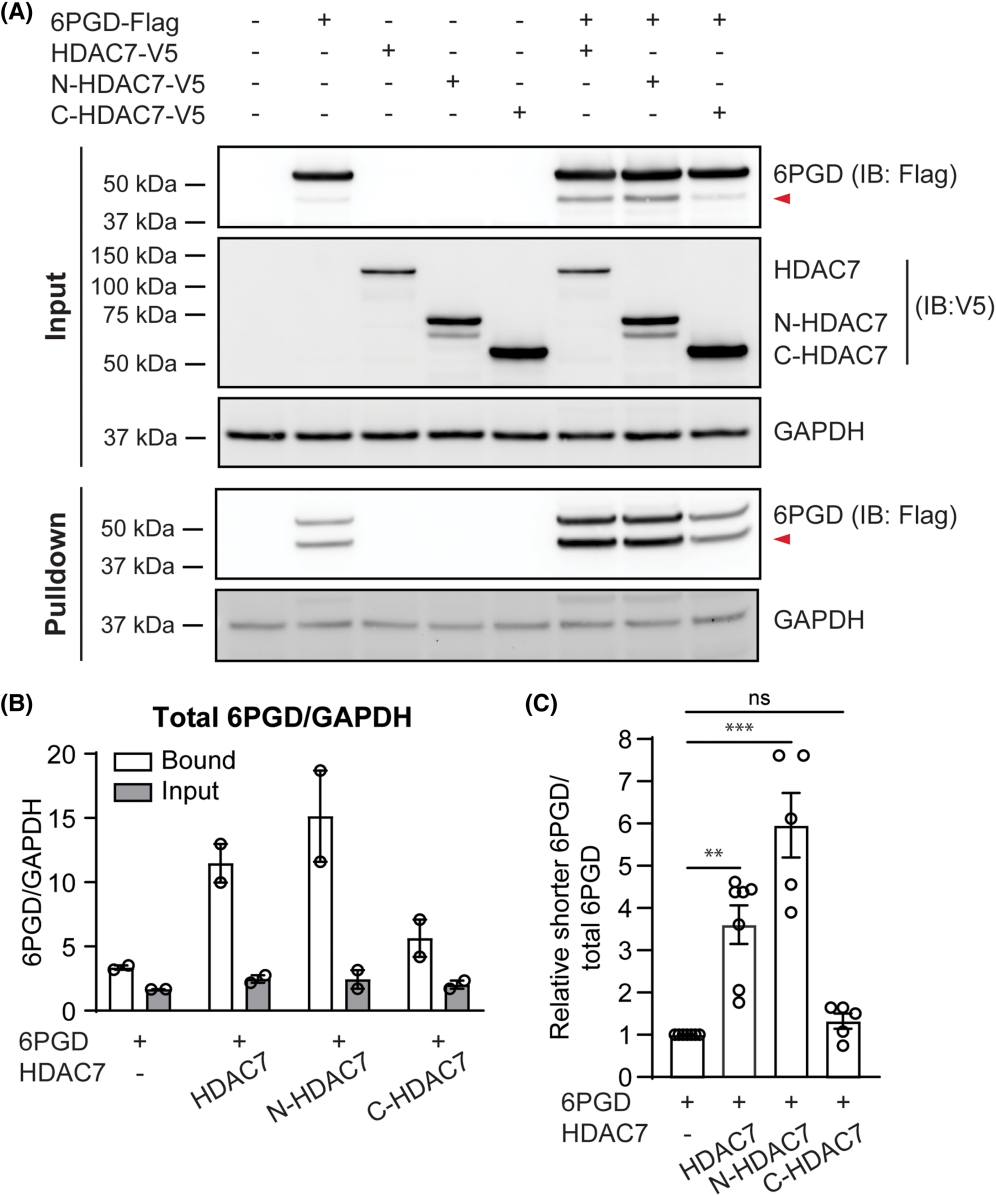
The N-terminus of HDAC7 enhances the affinity of 6PGD for NADP^+^. HEK293 cells were transfected with expression constructs encoding 6PGD (Flag-tagged), full-length HDAC7, N-HDAC7 and/or C-HDAC7 (V5-tagged) at a molar ratio of 1:1 (6PGD:HDAC) for 24 h. (**A**) Cells were then lysed, and the same amounts of protein lysates were used as input (top) and an affinity assay (bottom) using Cibacron Blue-conjugated agarose beads (100–200 mesh). Input lysates and agarose-precipitated proteins were then subjected to immunoblotting (anti-Flag, anti-V5) to assess levels of transfected proteins and the binding of 6PGD to Cibacron Blue. GAPDH was used as loading control. The displayed blots are from one experiment, representative of two independent experiments. The red arrow indicates the shorter form of 6PGD (6PGD-s). (**B**) Quantitative analysis of 6PGD levels relative to GAPDH. Total 6PGD levels were determined by quantifying the intensity of both 6PGD-s and full-length 6PGD in the input and Cibacron Blue pulldown samples, relative to respective GAPDH expression levels on immunoblots. Data are mean ± range from two independent experiments. (**C**) Quantitative analysis of 6PGD-s levels. Relative levels of 6PGD-s were quantified by comparing to the total combined band intensity of both 6PGD-s and full-length 6PGD on immunoblots of input lysates of HEK293 cells transfected with 6PGD and HDAC7, N-HDAC7 or C-HDAC7 from data presented in Figures 2, 3, 5 and 6. Data (mean ± SEM) are combined from 5 to 7 independent transfection experiments. Statistical analysis was performed using Kruskal–Wallis test followed by Dunn's multiple comparison (ns, not significant; **P < 0.01; ***P < 0.001).

6PGD uses NADP^+^ to generate NADPH in the PPP. In mouse macrophages, HDAC7 was required for the increase in the NADPH/NADP^+^ ratio in response to *Escherichia coli* infection [11]. Hence, we hypothesised that HDAC7 may increase the affinity of 6PGD for NADP^+^. To test this, we performed Cibacron Blue pulldown assays, since this dye has a high affinity for NAD^+^- and NADP^+^-binding proteins and has been used as an NADP^+^ mimic to study the substrate binding capacity of 6PGD [24] and other NADP^+^-binding proteins such as lactate dehydrogenase [29]. HEK293 cells were transfected with 6PGD and HDAC7 expression constructs, after which 6PGD and other NADP^+^-binding proteins were pulled down from cell lysates using Cibacron Blue-conjugated agarose beads. Levels of full length 6PGD were similar in all input samples (Figure 6A, top). However, more 6PGD was pulled down by Cibacron Blue-conjugated beads from cells overexpressing either WT or N-HDAC7 than 6PGD alone (Figure 6A, bottom; Figure 6B). The C-terminal deacetylase-containing domain of HDAC7, which did not promote 6PGD activation (Figure 4A,B), also did not lead to enrichment of 6PGD in the Cibacron Blue pulldown samples. In contrast to the effect of HDAC7 overexpression on 6PGD enrichment, similar levels of the NAD^+^-binding enzyme GAPDH [30] were pulled down in lysates from the various transfection conditions, suggesting that the N-terminus of HDAC7 selectively enhanced the NADP^+^-binding affinity of 6PGD. Interestingly, the shorter form of Flag-tagged 6PGD also bound to Cibacron Blue (Figure 6A, bottom, red arrow), with this binding being further increased by overexpression of WT HDAC7 or N-HDAC7 [compare the intensity of upper and lower bands in inputs (Figure 6A, top) versus pull downs (Figure 6A, bottom)]. These experiments suggest that the N-terminus of HDAC7 may increase 6PGD enzyme activity by enhancing NADP^+^ binding and that 6PGD-s likely has a higher affinity for NADP^+^. To further confirm the enrichment of 6PGD-s in cells expressing 6PGD-activating forms of HDAC7, its levels relative to total 6PGD (6PGD-s plus full-length 6PGD) were measured in cell lysates from the various co-transfection experiments. This analysis confirmed that both full-length HDAC7 and N-HDAC7 increased levels of 6PGD-s, whereas C-HDAC7 did not (Figure 6C).

### Multiple class IIa HDACs activate 6PGD

**Figure 7.**
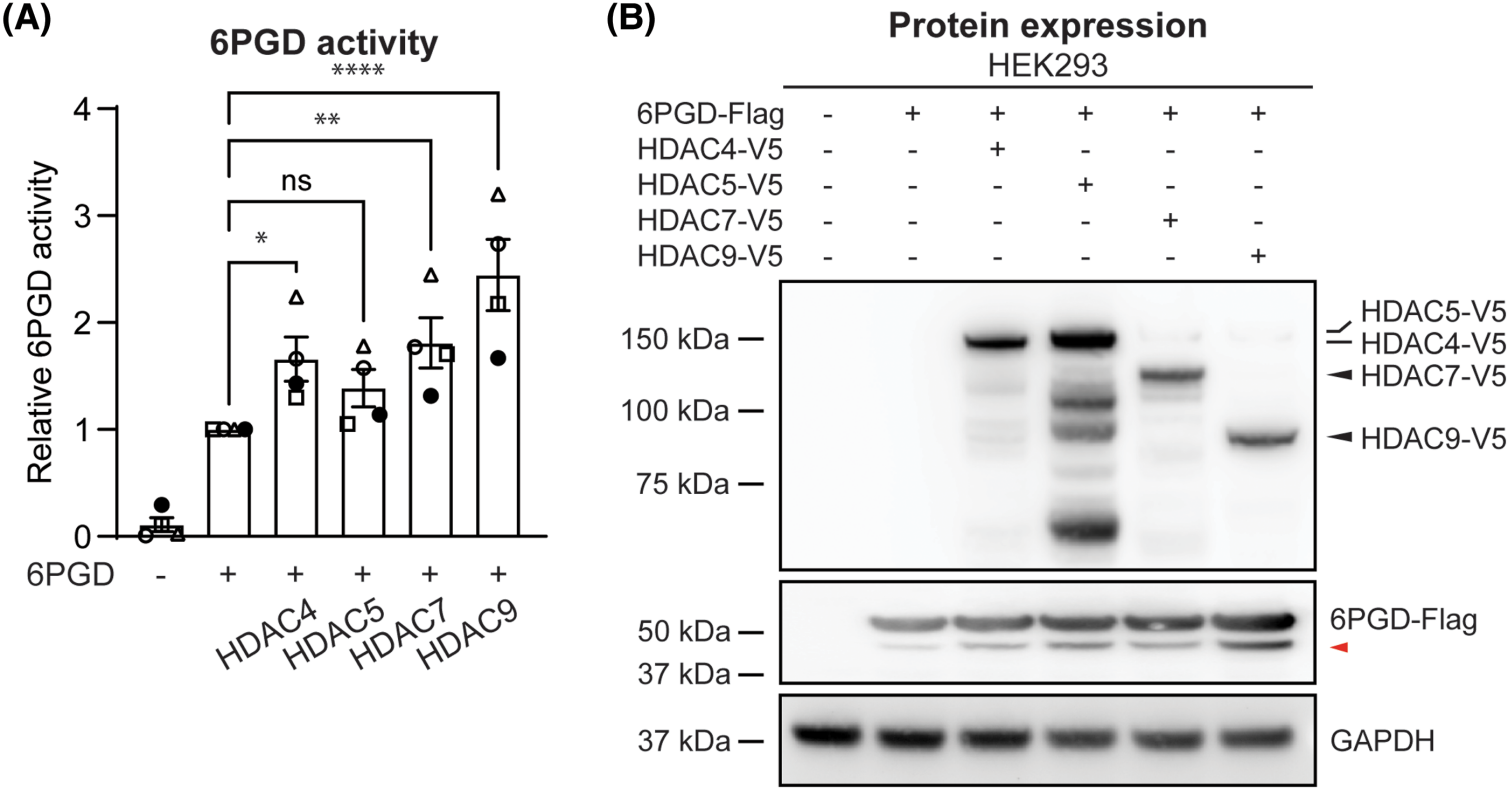
All class IIa HDACs activate 6PGD and induce production of a shorter form of 6PGD in transfected HEK293 cells. HEK293 cells were transfected with expression constructs encoding 6PGD (Flag-tagged) and class IIa HDACs (HDAC4, HDAC5, HDAC7 or HDAC9, V5-tagged) at a molar ratio of 1:1 (6PGD:HDAC), after which the enzymatic activity and levels of 6PGD in cell lysates was measured. (**A**) 6PGD enzymatic activity in HEK293 cells. Data (mean ± SEM) are combined from four independent experiments and are displayed as relative activity (normalised to levels in cells transfected with 6PGD alone). Statistical analysis was performed using repeated measure one-way ANOVA followed by Dunnett's multiple comparison (ns, not significant; *P < 0.05; **P < 0.01; ****P < 0.0001). (**B**) Protein expression of 6PGD (anti-Flag) and HDACs (anti-V5) in transfected HEK293 cells was assessed by immunoblotting, with GAPDH used as a loading control. The displayed blots are from one experiment, representative of two independent experiments. The red arrow indicates the shorter form of 6PGD (6PGD-s).

The observation that HDAC7 enzyme activity was not required for 6PGD activation was surprising, because we previously observed that the class IIa HDAC inhibitor TMP195 [31] attenuated *E. coli*-inducible 6PGD enzyme activity in human and mouse macrophages [11]. We considered that the effect of TMP195 may reflect involvement of multiple class IIa HDACs (HDAC4, 5, 7, 9) in 6PGD activation, since these four HDAC isozymes have a highly conserved deacetylase domain in their C-terminus. To test this, we compared the effect of other class IIa HDACs on 6PGD activity, transfecting HEK293 cells with expression constructs encoding 6PGD and the various class IIa HDACs. Here we observed that all class IIa HDACs (HDAC 4, 5, 7, 9) increased 6PGD activity in HEK293 cells, with HDAC9 inducing the highest 6PGD activation (Figure 7A). Thus, although HDAC7 is essential for maximal *E. coli*-induced 6PGD activation in macrophages [11], it is possible that other class IIa HDACs also contribute to this response. Interestingly, 6PGD-s was observed in all cells that were transfected with class IIa HDACs and its levels generally correlated with 6PGD enzyme activity (Figure 7B, red arrow). For example, HDAC9 showed the most pronounced effect in increasing 6PGD activation and resulted in the highest levels of 6PGD-s. These observations suggest that 6PGD-s may have increased enzymatic activity in HEK293 cells or that it may be generated upon 6PGD activation, for example as a proteolytic product.

## Discussion

HDAC7 promotes the PPP, antimicrobial ROS, and bacterial killing in macrophages, whilst also suppressing inflammatory responses in this setting [11]. This function relies, at least in part, on the interaction between HDAC7 and the PPP enzyme, 6PGD. However, the mechanisms by which HDAC7 activates 6PGD have not previously been reported. Reconstitution experiments in *Hdac7*^−/−^ macrophages confirmed the requirement for the enzymatic activity of HDAC7 in both bacterial uptake and killing by macrophages [11]. However, we show here that HDAC7 enhances 6PGD activity independently of its enzyme activity. It is possible that the enzyme-dependence for bacterial killing that was previously observed [11] reflects the role of HDAC7 enzyme activity in phagocytosis, rather than engagement of the PPP. Interestingly, the N-terminal region of HDAC7 lacking the deacetylase domain was sufficient to activate 6PGD in ectopic expression studies. Mechanistically, full-length HDAC7 and N-terminal HDAC7 did not obviously affect the dimerization of 6PGD but did facilitate the formation of higher order protein complexes and enhanced NADP^+^ binding, as assessed by Cibacron Blue pulldown assays. We also observed a shorter form of 6PGD (6PGD-s) that was selectively increased in cells overexpressing full-length HDAC7, the N-terminus of HDAC7 or other class IIa HDACs, all of which increased 6PGD activity. This suggests that HDAC7 may facilitate the cleavage of 6PGD to a more active form, although we cannot discount the possibility that 6PGD-s is generated as a feedback mechanism to inactivate 6PGD after full activation has been achieved.

We previously found that both the N-terminal and C-terminal domains of HDAC7 interact with 6PGD in a cell-free protein expression system [11]. Consistent with this, an interaction between 6PGD and both HDAC7 domains was detected in cells here (Figure 3). Although the C-terminus of HDAC7 did not increase the enzymatic activity of 6PGD (Figure 4), it may still play a role in regulating 6PGD functions. For example, it might permit trafficking of 6PGD to the phagosome for interaction with NOX2 [32]. If this is the case, the C-terminal domain of HDAC7 would be predicted to be important for 6PGD function even though it did not enhance 6PGD enzyme activity. Future investigations are warranted to study the localisation of 6PGD, HDAC7 and NOX2 in macrophages during phagocytic responses.

The N-terminal domain of HDAC7 possesses protein-binding motifs for the transcription factors CtBP and MEF2, as well as the scaffolding protein 14-3-3, enabling this lysine deacetylase to regulate signalling and gene expression through such interactions [3]. Overexpression of the N-terminus (amino acid 23-504) of HDAC7 in HEK293 cells increased 6PGD activity (Figure 4), suggesting that HDAC7 may enhance 6PGD activity via N-terminal domain-mediated scaffolding. Active 6PGD is a homodimer that generates both NADPH and ribulose-5-phosphate. Interestingly, the overexpression of HDAC7 or N-HDAC7 did not obviously affect 6PGD dimer formation. Rather, two higher order protein complexes were apparent in cells overexpressing either full-length HDAC7 or the N-terminus of HDAC7 (Figure 5A, right). These higher order complexes were detected in cells transfected with 6PGD alone or with 6PGD plus the negative control SOX7, implying that 6PGD interacts with unknown interaction partners in HEK293 cells and that HDAC7 further facilitates these interactions. Based on the estimated size of the protein complexes (∼150 and ∼250 kDa), potential candidates include the first and second PPP enzymes, G6PD and 6-phosphogluconolactonase. For example, dimeric 6PGD (∼100 kDa) could associate with tetrameric G6PD (∼150 kDa) [33] and/or monomeric 6-phosphogluconolactonase (∼30 kDa) [34] to generate a higher order complex to facilitate NADPH production. Another candidate is malic enzyme 1 (ME1), which scaffolds 6PGD in U2OS cancer cells, forming a hetero-oligomer of ∼150 kDa and enhancing its binding with 6-phosphogluconate [35].

Although HDAC7 enzyme activity was not required for 6PGD activation (Figure 1), it may facilitate other post-translational modifications on 6PGD to enable this response. While HDAC7 did not obviously facilitate the dimerization of 6PGD, Cibacron Blue pulldown assays that are used as a read-out for NADP^+^-binding [29] implicate the N-terminus of HDAC7 in increasing the NADP^+^ binding affinity of 6PGD (Figure 6). A previous study demonstrated that Fyn-dependent phosphorylation of 6PGD at tyrosine 481 (Y481) enhances its activity by increasing NADP^+^ binding [24]. It is therefore possible that scaffolding of HDAC7 to 6PGD via the HDAC7 N-terminus also permits 6PGD phosphorylation at Y481. In the human lung cancer epithelial-like cell line H1299, deacetylation of 6PGD at K76 and K294 by HDAC4 inactivated its activity [23]. In that study, the authors found that the HDAC inhibitors nicotinamide and Trichostatin A induced 6PGD activation in cancer cells. Using gene silencing in H1299 cells, HDAC4 and HDAC7 were identified as potential 6PGD deacetylases; however, only HDAC4 was implicated in direct deacetylation of recombinant 6PGD. Taken together with our findings that HDAC7 activates 6PGD in primary mouse macrophages [11] and that multiple class IIa HDACs activate 6PGD in HEK293 cells (Figure 7), it is likely that there are signal- and/or cell type-specific mechanisms by which these lysine deacetylases regulate 6PGD activity. This would be consistent with the fact that the PPP has cell-specific functions, enabling cell proliferation in cancer cells [36], rapid ROS production for bacterial killing in innate immune cells [37], and regulatory control of T cell development and effector functions [38,39].

In HEK293 cells overexpressing 6PGD, 6PGD-s was increased in conditions where 6PGD enzyme was most active, namely in cells transfected with full-length HDAC7, N-HDAC7 or other class IIa HDACs. Presuming this occurs through proteolytic cleavage, it is estimated that 6PGD-s lacks the N-terminal 70–80 amino acids. The NADP^+^ binding affinity of 6PGD-s was increased by HDAC7 (Figure 6A), suggestive of increased enzymatic activity. Several residues in 6PGD that are important for NADP^+^ binding have been identified. These include K76 near the N-terminus [23], Y481 towards the C-terminus [24] and several other residues in the dimeric interface that are involved in both NADP^+^ and 6-phosphogluconate binding [40]. The nature of 6PGD-s, whether it homodimerizes or heterodimerizes with full-length 6PGD, and how it affects 6PGD enzyme activity and function remain open questions. However, a shorter form of 6PGD was also observable in some blots from a previous study in which 6PGD was co-expressed with ME1 in 293T cells [35]. Given the likely increased affinity of 6PGD-s for NADP^+^ (Figure 6A), it is possible that ME1 and HDAC7 both enhance 6PGD activation by promoting the generation of 6PGD-s. Interestingly, 6PGD-s was not detected when lysates from cells transfected with HDAC7 or N-HDAC7 were crosslinked (Figure 5A, right). This suggests that 6PGD-s may homodimerize or form a heterodimer with full-length 6PGD to generate its active form(s). Although the exact sequence of 6PGD-s remains to be determined, we speculate that cleavage of an N-terminal region exposes the 6-phosphogluconate and/or NADP^+^ binding pockets in 6PGD-s to facilitate substrate and/or co-factor binding.

**Figure 8.**
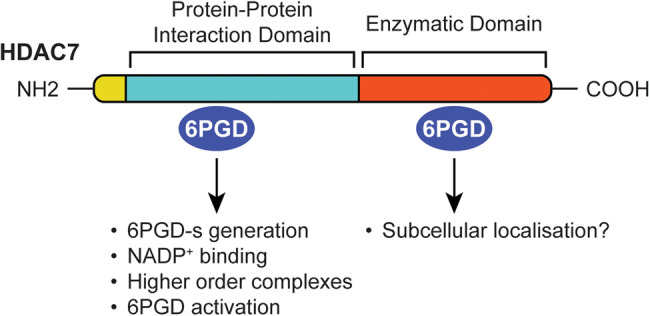
Model of HDAC7 involvement in 6PGD functions. The N-terminal protein-protein interaction domain of HDAC7 interacts with 6PGD, leading to the generation of a short form of 6PGD-s, increased NADP^+^ binding to 6PGD, formation of higher order 6PGD-containing complexes, and increased 6PGD enzyme activity. The C-terminal enzymatic domain of HDAC7 also interacts with 6PGD but does not elicit these effects. It is possible that it exerts other effects that are required for 6PGD functions in cells, for example control of subcellular localisation.

In summary, we have provided insights into mechanisms by which HDAC7 activates the PPP enzyme 6PGD. HDAC7 binds 6PGD and promotes its enzymatic activity independently of HDAC7 enzymatic activity, acting instead via the N-terminal protein-protein interaction domain (Figure 8). The interaction between the N-terminal domain of HDAC7 and 6PGD likely increases the binding affinity of 6PGD for NADP^+^. We previously reported antibacterial and anti-inflammatory roles for HDAC7 in myeloid cells responding to bacterial challenge, with this involving HDAC7-mediated 6PGD activation [11]. Although the C-terminal domain of HDAC7 did not increase 6PGD activation in ectopic expression studies, it did interact with 6PGD in cells (Figure 3) and when expressed in a cell-free system [11]. Moreover, both genetic deletion of *Hdac7* and pharmacological inhibition of class IIa HDACs attenuated *E. coli*-induced 6PGD enzyme activity in macrophages [11]. We therefore propose that HDAC7 has multiple roles in inducible 6PGD activation in macrophages and/or that other class IIa HDACs are also involved in this response. Future studies should address how the N-terminal protein interaction domain of HDAC7 influences 6PGD activation, flux through the PPP, and antimicrobial ROS production in macrophages, as well as the role of HDAC7 enzyme activity in these processes. Such information can guide our understanding of HDAC7 functions in different cellular contexts and inform strategies for manipulating enzymatic and non-enzymatic activities of this protein in inflammatory and/or infectious diseases. Findings reported here may also inform strategies for boosting 6PGD functions to suppress inflammation in the context of chronic disease.

## Materials and methods

### Cell culture and transfection

HEK293 cells were cultured in DMEM (Gibco) supplemented with 10% foetal bovine serum, 50 U/ml penicillin and 50 μg/ml streptomycin (Gibco) at 37°C in humidified 5% CO_2_ ventilated air. To overexpress proteins, HEK293 cells were transiently transfected with expression constructs encoding mouse 6PGD-Flag (pCMV-based) and pEF6-based constructs (C-terminal V5 tag) encoding mouse HDAC6, HDAC7, HDAC7 mutants (N-HDAC7, C-HDAC7, ED-HDAC7), HDAC4, HDAC5, HDAC9, and/or SOX7 (unrelated control protein). Briefly, HEK293 cells were plated in 6-well (0.8 × 10^6^ cells) or 12-well (0.5 × 10^6^ cells) plates and incubated overnight. The Lipofectamine™ 2000 (Invitrogen, 11668019) transfection reagent was prepared in OptiMEM with DNA at a final concentration of 1 μg/μl DNA. Plasmids were diluted in OptiMEM (Gibco) and co-transfections were performed with 1:1 molar equivalents, with the total amount of plasmid used for each transfection condition being kept constant by adding the pEF6 empty vector. Prior to transfection, cell culture media was replaced with serum- and antibiotic-free OptiMEM, the transfection mixture was then added into cells for 6–8 h before being supplemented with fresh cell culture medium. At 24 h post-transfection, culture supernatants were discarded, and cells were briefly washed with DPBS (Gibco) and lysed with respective buffers for different analyses.

### 6PGD activity assays

HEK293 cells were transfected with a total amount of 2 μg of indicated constructs for 24 h and lysed in 6PGD assay buffer (Abcam, ab241016). Total protein concentration was quantified using a Pierce BCA Assay kit (Thermo Scientific, 23225), following the manufacturer's instructions. Equal amounts of cell lysates from each condition were subjected to 6PGD activity assays using an *in vitro* assay (Abcam), as per the manufacturer's instructions. The enzymatic activity was determined by measuring absorbance at 460 nm in kinetic mode with a plate reader (Infinite M Plex, Tecan). 6PGD activity was calculated by determining the amount of NADPH generated between two timepoints, with the use of a standard curve.

### Class IIa HDAC activity assays

Transfected cells were lysed in 6PGD assay lysis buffer and 10 μl of lysates containing the same amount of protein were transferred to a 96-well plate for quantification of class IIa HDAC enzyme activity. 200 μM class IIa HDAC substrate BOC-Lys(trifluoroacetyl)-AMC [31] (manufactured in-house) dissolved in 50 μl assay buffer (50 mM Tris HCl, 137 mM NaCl, 2.7 mM KCl and 1 mM MgCl_2_, pH 8.0) was added to the lysates and incubated at 37°C for 30–60 min. The enzymatic reaction was quenched using 50 μl stop solution containing 20 mM SAHA (manufactured in-house) and 1 mg/ml trypsin (Invitrogen) in assay buffer. Fluorescence intensity was measured at excitation and emission wavelengths of 350 and 460 nm, respectively, using a plate reader (Infinite M Plex, Tecan). Enzymatic activity was expressed as fluorescence intensity.

### Co-immunoprecipitation experiments

HEK293 cells were transfected with 6 μg of indicated expression constructs for 24 h, after which cells were briefly washed with DPBS and lysed in RIPA buffer (50 mM Tris-Cl, 150 mM NaCl, 0.1% SDS, 1% sodium deoxycholate, 1% NP-40) or mild lysis buffer (50 mM HEPES, 150 mM NaCl, 1.5 mM MgCl_2_, 1 mM EDTA, 10% glycerol and 1% Triton X-100) supplemented with 1 X protease inhibitor cocktail (cOmplete™, Roche, 11873580001) and 1 X phosphatase inhibitor (PhosSTOP™, Roche, 4906837001). DNA within cell lysates was digested with 1 μl benzonase (Millipore, E1014) at room temperature for 20 min. The soluble phase was then collected as the input, which was then subjected to immunoprecipitation. Input lysates were incubated with 1 μg of bait antibody (mouse anti-V5, Bio-Rad, MCA1360; mouse anti-Flag, Sigma-Aldrich, F3165; rabbit anti-Flag, Cell Signaling Technology, 14793) on a rotator at 4°C overnight or at RT for 2 h, after which 35 μl Dynabeads Protein G (Invitrogen, 10004D) were suspended in lysis buffer and added into the samples. The immunoprecipitation was performed as per the manufacturer's instructions using a DynaMag™ Magnet (Invitrogen). Briefly, the magnetic bead-antibody-protein complex was washed in lysis buffer or DPBS three times. Precipitated proteins were eluted in NuPAGE loading buffer (Invitrogen) containing reducing agent (Invitrogen) and boiled before being analysed by immunoblotting.

### Crosslinking assays

Transfected HEK293 cells were washed once with DPBS and lysed in lysis buffer (50 mM HEPES, 150 mM NaCl and 0.5% Triton X-100) containing 1 X protease inhibitor cocktail (Roche) and 1 X phosphatase inhibitor (PhosSTOP™, Roche). The soluble phase was then collected and incubated with DSS (Thermo Fisher) at a concentration of 2 mM for 30 min or 0.025% glutaraldehyde for 15 min at room temperature. Crosslinking reactions were quenched with 50 mM Tris-HCl at room temperature for 15 min. Protein concentrations were quantified using BCA assays and equal amounts of samples were subjected to analysis by immunoblotting.

### NADP^+^ binding assays

The NADP^+^ affinity of 6PGD was assessed using Cibacron Blue F3GA covalently-attached beads (Bio-Rad, 1537302). Transfected HEK293 cells were lysed in lysis buffer (50 mM HEPES, 150 mM NaCl, 1.5 mM MgCl_2_, 1 mM EDTA, 10% glycerol and 1% Triton X-100) supplemented with 1 X protease inhibitor cocktail (Roche) and 1 X phosphatase inhibitor (PhosSTOP™, Roche). The soluble phase was collected and quantified using BCA assays. Equal amounts of each sample were incubated with 20 μl Cibacron Blue F3GA agarose beads on a rotator at 4°C for 2 h. Following the incubation, beads were washed three times with lysis buffer, eluted and boiled in NuPAGE loading buffer containing reducing agent. Eluted samples and input lysates were then subjected to immunoblotting as an indirect read-out of NADP^+^ binding capacity.

### Immunoblotting

To analyse protein levels, cell lysates were subjected to electrophoresis using pre-cast 4–12% Bis-Tris NuPAGE gels (Invitrogen). After samples were separated, proteins were transferred to a nitrocellulose membrane (Bio-Rad) using a Trans-Turbo Blot system (Bio-Rad) and blocked with 5% skim milk in Tris-buffered saline (TBS, 10-mM Tris-HCl pH 8.0, 150-mM NaCl) with 0.05% Tween-20 (TBST). Membranes were then probed with anti-Flag (mouse anti-Flag, 4 μg/ml, Sigma-Aldrich, F3165; rabbit anti-Flag, 0.8 μg/ml, Sigma-Aldrich, F7425; rabbit anti-Flag, 0.414 μg/ml, Cell Signaling Technology, 14793) or anti-V5 antibodies (mouse anti-V5, 1 μg/ml Bio-Rad, MCA1360; rabbit anti-V5, 0.192 μg/ml, Cell Signaling Technology, 13202) at 4°C overnight. HRP-conjugated (1/2500 dilution, Cell Signaling Technology, 7074, 7076) or Starbright blue (1/2500 dilution, Bio-Rad, 12004161, 12005866) anti-rabbit and anti-mouse antibodies were used as secondary antibodies. Rhodamine-conjugated antibodies (1/5000 or 1/10 000 dilution, Bio-rad, 12004165, 12004167) against Tubulin and GAPDH (used as loading controls) were also added onto membranes in this step. Membranes were then visualised after adding Immobilon Western Chemiluminescent HRP substrate (Millipore) for HRP-conjugated antibodies or immediately after washing for fluorescent antibodies using a ChemiDoc MP System (Bio-Rad). Protein quantification was performed using the Image Lab Software (Bio-Rad).

### Statistical analyses

Experimental data were combined from independent experiments, with the average from technical duplicates or triplicates within each experiment being used to compile data for statistical analyses. Data shown are mean and error bars indicate range or standard error of the mean (SEM), as indicated in individual figure legends. Statistical analyses were performed using Prism 9 and 10 (GraphPad), with individual statistical tests described in the figure legends. Difference with confidence value of 95% (*P* < 0.05) were considered as statistically significant.

## Data Availability

All data from this study are included in the article.

## Open Access

Open access for this article was enabled by the participation of University of Queensland in an all-inclusive *Read & Publish* agreement with Portland Press and the Biochemical Society under a transformative agreement with CAUL.

## Abbreviations

DSS: disuccinimidyl suberate
ED: enzyme dead
EV: empty vector
G6PD: glucose-6-phosphate dehydrogenase
HDAC: histone deacetylases
LPS: lipopolysaccharide
ME1: malic enzyme 1
NADPH: nicotinamide adenine dinucleotide phosphate
6PGD: 6-phosphogluconate dehydrogenase
PKM2: pyruvate kinase isoform M2
PPP: pentose phosphate pathway
RL5P: ribulose-5-phosphate
ROS: reactive oxygen species
SEM: standard error of the mean
WT: wild type
